# TFC‐1326 Compound Reduces Clinical Signs of Skin Aging. Evidence From In Vitro Human Adipose and Skin Models and Pilot Clinical Trial

**DOI:** 10.1111/jocd.16679

**Published:** 2024-11-17

**Authors:** Géraldine Deliencourt‐Godefroy, Jocelyne Legoedec, Marion Bourdens, Noémie Juin, Linh‐Trang Nguyen, Marie‐Christine Branchet, Sylvie Boisnic, Mayoura Keophiphath

**Affiliations:** ^1^ TFChem (Sirona Biochem Corp) Val‐de‐Reuil France; ^2^ DIVA Expertise Toulouse France; ^3^ Gredeco Paris France

**Keywords:** anti‐freeze glycoproteins, dermis density, hypodermis, inflammageing, matrix remodeling, wrinkles

## Abstract

**Background:**

Anti‐freeze Glycoproteins (AFGPs) were described to preserve biological materials and protect them from different stresses.

**Aims:**

The effects of a synthetic anti‐freeze glycoprotein‐based compound, TFC‐1326, on human skin quality and its biological actions were studied.

**Methods:**

The effects of various concentrations of TFC‐1326 on the biology of human preadipocytes, differentiated in the proinflammatory microenvironment, and on human fibroblasts grown in coculture with human mature adipocytes or monocultured in stress conditions were investigated in, in vitro studies. Additionally, the efficacy of a 1% TFC‐1326 topical cream was evaluated in a clinical investigation on the skin biology and appearance of 20 women aged between 40 and 65 years throughout 84 days of application.

**Results:**

The in vitro studies revealed that TFC‐1326 mitigated the deleterious effects of a proinflammatory cytokine cocktail produced by human macrophages, by restoring preadipocyte adipogenic capacity and by reducing their fibroinflammatory state. TFC‐1326 also stimulated the proliferative capacity of dermal fibroblasts co‐cultured with mature adipocytes as well as their production of hyaluronic acid and procollagen type I, while decreasing IL6 secretion and increasing fibroblast viability. Furthermore, daily 1% TFC‐1326 topical cream application, measurably improved skin radiance and laxity, as well as skin density. Finally, significant reductions of the volume and depth of the crow's feet wrinkles were also observed.

**Conclusions:**

The compound TFC‐1326 significantly improved the physiological appearance and cellular functions of aging skin.

## 1 Introduction

**FIGURE 1:**
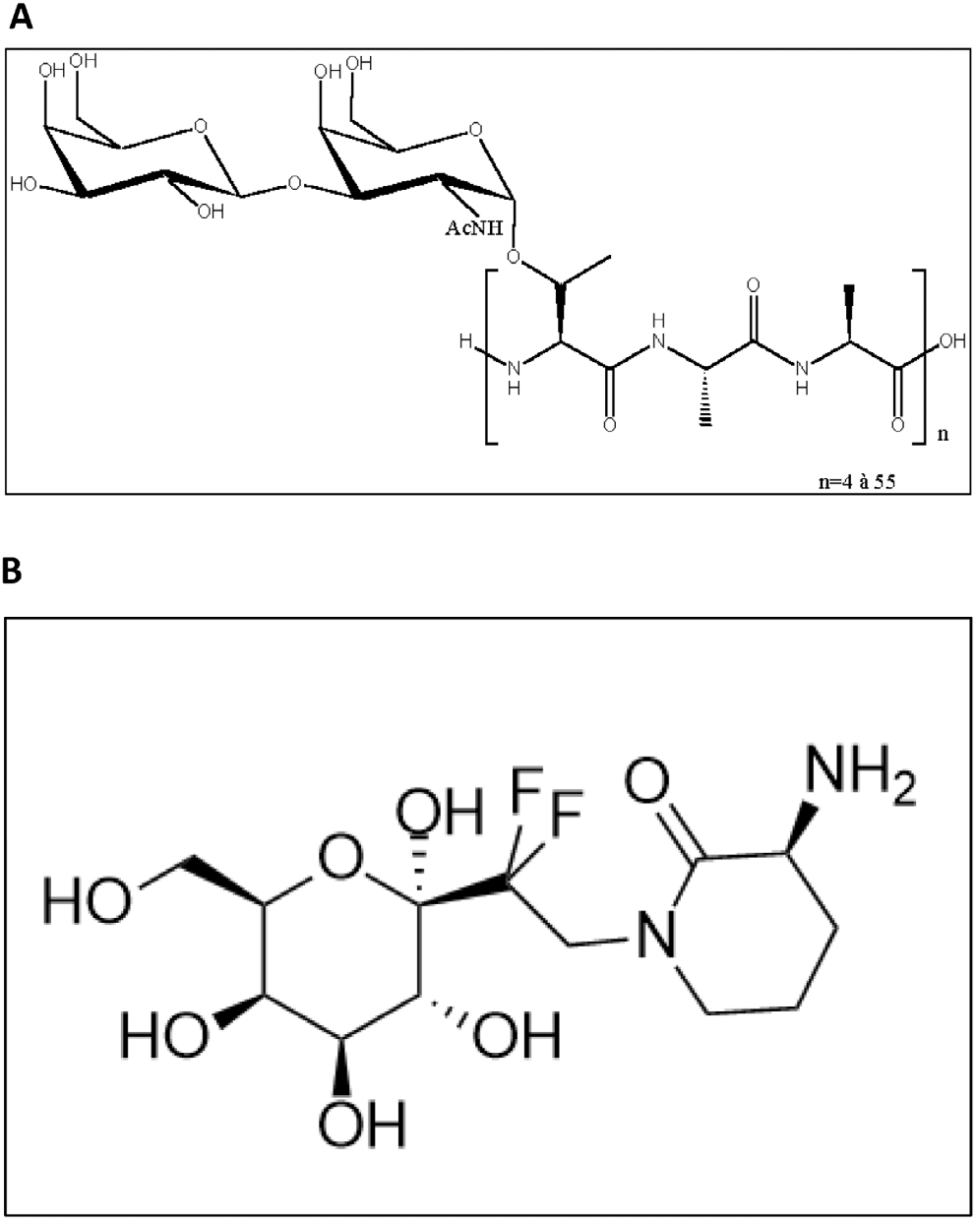
Chemical structures of natural antifreeze glycoproteins (A) and of TFC‐1326 (B).

Nature and how it resolves challenges has always been a source of inspiration for scientists. Then, biomimicry became a major provider of innovation to develop pharmaceutical or cosmetic actives [1, 2, 3]. Antifreeze Glycoproteins (AFGPs) were known for their potential to preserve biological materials (cells, tissues, and organs). AFGPs have an unusual chemical structure, which consists of repeating units (between 4 and 50 times) of three amino acids (Ala‐Ala‐Thr) and the disaccharide β‐D‐galactosyl‐(1 → 3)‐α‐N‐acetyl‐D‐galactosamine joined as a glycoside to the hydroxyl oxygen of the Thr residues (Figure 1A). They were discovered in 1957 in polar fish and constitute the major fraction of the different proteins in their blood serum [4]. They have the property to protect these species against stress and especially cold damage [5]. They lower the freezing point of body fluids in a non‐colligative manner, a process termed thermal hysteresis, and inhibit ice recrystallization during rewarming of frozen solutions. In addition, they enhance membrane stability during stresses such as low temperatures and freezing conditions [6, 7]. Consequently, AFGPs would be useful as safe cryoprotectants in maintaining whole human organs for transplant without time constraints, preserving delicate tissues or cells with minimal damages [5, 8, 9, 10]. We were interested in developing one of the stress protective effects of AFGP, namely, against oxidative stress.

Firstly, we focused our work on this class of compounds to mimic them in order to obtain stable compounds and to avoid possible beta‐elimination [11, 12], and hydrolysis that may occur on the anomeric position of the sugar moiety. In addition, we focused our work on the monomeric unit (*n* = 1) (Figure 1A), which is not required for the antifreeze action, where polymeric structures are involved [13]. We were able to successfully build a family of small derivatives (1 sugar +3 to 1 amino acid) with improved stability and efficacy, amongst which was TFC‐1326 (Figure 1B). TFC‐1326 showed a promising profile for cosmetic applications, which went beyond just protective effects alone [14]. Its chemical structure was different from the other members of the family, and this difference could have advantages for cosmetic applications by allowing better permeation. Furthermore, the stress‐protective effects of this family of compounds can be highly beneficial in preventing skin aging. In addition to the efficacy of this new molecule, the presence of CF_2_ prevents any breakdown of the aglycone part, solving the instability issue of the natural compound. Its INCI name is Galactose Difluoroethyl Aminopiperidinone.

Many countries in the world are experiencing increases in both the number and proportion of older people in their population. In this context, the challenge is to maintain health into old age, and one of the strategies is to reduce the chronic and low‐grade inflammation associated with aging. The increased levels of circulating proinflammatory factors are largely shown to be related to alterations in whole‐body organs and tissues, and the skin is not spared [15]. This “inflammageing” was described as contributing to and/or accentuating the clinical signs of aging, including wrinkles, relaxation, and dryness of the skin, mainly as a result of reduced production of Extracellular Matrix (ECM) proteins and from organizational changes [16, 17]. The human skin is composed of three layers, from the outside to the inside: epidermis, dermis and hypodermis [18]. Several studies have shown that the hypodermis is a dynamic tissue, displaying changes in size and in metabolism throughout life. The hypodermis corresponds to the adipose compartment of the skin and is a specific subcutaneous layer intimately underlying the reticular dermis. More than a structural support, the hypodermis, through its endocrine function, plays crucial roles in the regulation of numerous physiopathological processes like hair follicle cycling, wound healing, and skin aging and is involved in epidermal and dermal homeostasis [19]. Alterations in hypodermal adipose tissue, probably linked to local inflammation, may support clinical manifestations of aging skins [20]. As 80% of the visible signs of skin aging, including facial fine lines, winkles, loose and sagging skin, are due to external factors such as UV, pollution, tobacco, etc., there is a great need for compounds that can protect the skin from external stresses [21].

In this article, we aimed to highlight new potential anti‐aging effects of TFC‐1326 by presenting results from preclinical studies and from one pilot clinical trial. The primary outcome of the clinical trial was to confirm the stress‐protective effect of a simple compound containing TFC‐1326 as an active ingredient, especially against inflammation and oxidation. The secondary aims were to assess how this impacted overall rejuvenation, including improvements in skin texture (density, radiance), skin sagging, and wrinkle reduction.

## 2 Materials and Methods

### 2.1 Synthesis of TFC‐1326

Details of the synthesis of the compound is described in patent WO2022/157233A1 [14].

### 2.2 Cellular Studies

#### 2.2.1 Isolation of Human Fibroblasts, Preadipocytes and Mature Adipocytes

Skin and subcutaneous adipose tissue biopsies were obtained from 4 non‐obese (body mass index 25.15 ± 4.32 kg/m^2^) and young (41 ± 11.8 years old) female patients undergoing abdominal aesthetic or reconstructive surgery. This study was approved by the bioethical unit of the French Ministry of higher education, research, and innovation (Authorization number: AC‐2023‐6079 and declaration number: DC‐2016‐2853).

Human fibroblasts were prepared from an abdominal skin biopsy taken from a 56‐year‐old donor with a BMI of 28 kg/m^2^. The skin was degreased before being cut into small explants that then cultured in petri dishes containing DMEM medium enriched with 20% fetal bovine serum (FBS) + 1% penicillin–streptomycin +1% fungizone and incubated at 37°C and 5% CO_2_ for at least two weeks.

Human preadipocytes were isolated and cultured as previously described [22]. Briefly, minced adipose tissue was digested with collagenase while stirring. The digested material was filtered and centrifuged. The resulting pellet (stroma vascular fraction, SVF) was washed and resuspended in DMEM +10% FBS + 1% penicillin–streptomycin + 1% fungizone before seeding and culturing in flasks incubated at 37°C and 5% CO_2_ for at least one week to obtain preadipocytes.

The formation of adipocyte capsules followed an internally standardized protocol [23]. Briefly, mature adipocytes were prepared from the digestion of adipose tissue with collagenase for 30 min under gentle agitation. After several washes, mature adipocytes were encapsulated in a hydrogel to form 3D adipocyte capsules. The capsules of mature adipocytes were incubated in the DMEM culture medium containing bovine serum albumin and insulin, at 37°C and 5% CO_2_.

#### 2.2.2 Irradiation and FBS Starvation Stress on Dermal Fibroblasts

For irradiation experiments, human dermal fibroblasts (HDF) were seeded in 96‐well plates and cultured in culture medium (DMEM supplemented with L‐glutamine 2 mM, Penicillin 50 U/mL, Streptomycin 50 μg/mL, FBS 10%) for 24 h. The medium was then replaced by culture medium containing or not the tested product, and the cells were pre‐incubated for 24 h. After pre‐incubation, the medium was removed and replaced by a specific irradiation medium (EBSS supplemented with CaCl_2_ 0.2 g/L, MgSO_4_ 0.2 g/L), and the cells were irradiated at 35 J/cm^2^. The lamp used was a SOL500 Sun Simulator equipped with an H1 filter (Dr. Hönle, AG). After irradiation, the medium was removed and replaced by DMEM medium containing or not the tested product and the cells were incubated for 24 h. A non‐irradiated control condition was set up in parallel. All experimental conditions were performed in quintuplicate of culture (*n* = 5), except for the control conditions in *n* = 12. The product was tested at concentrations of 1.25 and 2.25 mg/mL.

For FBS starvation stress experiments, human dermal fibroblasts were cultivated without FBS to induce starvation stress and were supplemented with or without the product at 6 mg/mL for 7 days of culture.

#### 2.2.3 Differentiation of Human Preadipocytes in Proinflammatory Conditions

Preadipocytes were seeded and incubated in 100 μL of DMEM +10% FBS in 96‐well plates. The following day, they were treated with the tested product at three doses (1, 2, and 5 mg/mL) in a proadipogenic cocktail containing insulin, glucocorticoid, 3‐isobutyl‐1‐methylxanthine (IBMX), and thiazolinedione. To induce proinflammatory conditions, cells were simultaneously treated with an activated human macrophage‐conditioned medium (ACMC) for 14 days. ACMC was prepared according to an internal procedure described previously [24, 25]. The medium was changed every 3 days for up to 14 days. During the last 24 h of culture, the cells were incubated in fresh DMEM/F12 medium alone to collect cellular secretions. Dexamethasone (DEXA), which is a synthetic glucocorticoid, was used at a concentration of 100 nM as an anti‐inflammatory control. All experimental conditions were performed in triplicate of culture.

#### 2.2.4 Coculture of Human Dermal Fibroblasts and Mature Adipocytes

Dermal fibroblasts were seeded and incubated in DMEM with 10% FBS in 96 well plates. The day after, the floating adipocyte capsules were co‐cultured in suspension above the fibroblasts, and the medium was changed and replaced by a specific culture medium optimized for co‐culture of these two cell types. Treatment with the product at 3 concentrations (1, 2, and 3 mg/mL) was initiated at day zero (D0), with medium changes at D2, D3, and D5. The secretion media from 48 h incubations were collected at D2 and D5, and the secretion media from 24 h incubations were collected at D3 and D6, before being centrifuged and stored at −80°C for further analyses. On the last day of treatment (D6), cells corresponding to positive control of cytotoxicity were treated with 1% of triton for 4 h. After medium collection, fibroblasts and adipocytes were fixed with a paraformaldehyde solution (PFA 4%) and stored in PBS solution at +4°C until immunostaining experiments. Each experimental condition was done in triplicate of culture.

#### 2.2.5 Evaluation of Fibroblast Viability

At the end of the culture period in various stress conditions, the fibroblasts were incubated with MTT (solution of tetrazolium salt) reduced to blue formazan crystals by succinate dehydrogenase (mitochondrial enzyme). This transformation is proportional to the enzyme activity. After cell dissociation and formazan crystal solubilization using DMSO, the optical density (OD) of the extracts at 540 nm, which is proportional to the number of living cells and their metabolic activity, was recorded with a microplate reader (VERSAmax, Molecular Devices). Cell viability was evaluated at day 3 or at day 0, day 4, and day 7, respectively, for irradiation and FBS starvation experiments.

#### 2.2.6 Quantification of Cell Number and Lipid Accumulation in Preadipocytes

After 14 days of culture, fixed preadipocytes were stained by AdipoRed and DAPI (4′,6‐Diamidino‐2‐Phenylindole, Dihydrochloride) (D1306, Molecular Probes) at room temperature to reveal the intracellular lipid droplets and the nuclei, respectively. Quantification of lipid accumulation and cell number were performed by automated fluorescence measurements and an algorithm detecting specifically the lipid droplets and the nuclei (EJON). Data are represented using a Lipid accumulation Index (area × intensity of the AdipoRed staining) and normalized to cell number [26].

#### 2.2.7 Immunofluorescence and Quantification of Collagen I in Preadipocytes and Fibroblasts

After fixation of preadipocytes or fibroblasts, cells were incubated with 3% Bovine Serum Albumin (BSA) for 30 min to block their non‐specific sites, then with anti‐collagen I primary antibody (Novusbio, NB600‐408) over night. After washes with PBS solution, cells were incubated for 30 min with 3% BSA solution and then with secondary antibodies (Goat anti‐rabbit alexa‐fluor 488, ThermoFisher, A11008) and DAPI (nuclei stain) for 1 h. Following several washes, acquisition and quantification were carried out using a fluorescent videomicroscope. Briefly, the quantification was based on the detection and quantification of cell nuclei stained with DAPI and the detection of collagen I staining by two methods: Collagen I fibers were detected and measured for their length, thickness, and intensity. The quantity of Collagen I fibers was calculated (Quantity = length × thickness × fluorescence intensity) and normalized to cell number. An overall Collagen I (neosynthesized collagen I) considered as a non‐fibrillar signal was detected, measured, and normalized to cell number.

#### 2.2.8 Quantification of Extracellular Secretions

The biochemical analyses on culture media of cells were performed via ELISA using specific kits according to the manufacturer's recommendations: IL‐6 (Duoset DY206, R&D Systems), MCP1 (Duoset, DY279‐05, R&D Systems), Procollagen I (Duoset, DY6220‐05, R&D Systems), and Hyaluronic Acid (HA) (Duoset, DY3614‐05, R&D Systems). Values were normalized to cell number.

Cytotoxicity was measured by the lactate dehydrogenase activity (G1780 Promega) according to the manufacturer's instructions. All the biochemical results were represented in percentage of the positive control of cytotoxicity.

### 2.3 Clinical Study

This open study was performed under dermatological control. The subjects were their own references, and a comparison was made with starting values. The clinical study was conducted in accordance with the Declaration of Helsinki, and the preclinical studies were approved by the bioethical unit of the French Ministry of higher education, research, and innovation (Authorization number: AC‐2023‐6079 and declaration number: DC‐2016‐2853). Informed consent was obtained from all subjects involved in the clinical study and also from all the donors of skin and adipose tissues used in the preclinical studies (surgical wastes). Written informed consent was also obtained from the patients to publish this paper.

Since this clinical monocentric open‐labeled study was carried out on healthy volunteers, it was not considered “Research Involving the Human Person” because it was not carried out with a view to the “development of medical or biological knowledge,” as described in French Decree no. “2017‐884 of May 9, 2017.”

#### 2.3.1 Studied Population

As we collected mainly abdominoplasties from aesthetic surgeries on women patients, we continued the clinical study on the same gender in order to homogenize as much as possible and to limit the variability of the results. Twenty healthy Caucasian women between 40 and 65 years old (mean age: 55.75 ± 5.57 years) showing signs of aging, such as a sagging of the lower face associated with nasolabial fold wrinkles, were included in the study following verification of inclusion and exclusion criteria. Exclusion criteria were unhealthy subjects or those presenting dermatological or connective tissue pathologies. Additional exclusion criteria were pregnant and breastfeeding women. Subjects were asked to maintain their cosmetic habits throughout the study.

#### 2.3.2 Product Composition

The composition of the Topical cream TFC‐1326 1% is described in Table 1.

**TABLE 1:** Composition of the topical cream containing 1% of TFC‐1326.

Ingredient list of the topical cream
AQUA (WATER), GLYCERIN, ISOAMYL LAURATE, BUTYROSPERMUM PARKII (SHEA) BUTTER, *Prunus ARMENIACA* (APRICOT) KERNEL OIL, C12‐16 ALCOHOLS, BEHENYL ALCOHOL, TFC‐1326, PALMITIC ACID, HYDROGENATED LECITHIN, XANTHAN GUM, CITRIC ACID, PARFUM (FRAGRANCE), SODIUM BENZOATE, POTASSIUM SORBATE, SODIUM GLUCONATE, SODIUM HYDROXIDE

#### 2.3.3 Experimental Plan

The product was applied twice daily on the entire face, once in the morning and once in the evening, for 84 days. Evaluations were carried out at D0, D56, and D84 on each of the included patients. In total, 3 visits were made over a three‐month period of treatment in order to follow the evolution of the different parameters: skin radiance, oxidation, inflammation, density, winkles, and oval face sagging.

#### 2.3.4 Colorimetric Representation and Volume Measurement of the Oval of the Face

Colorimetric representations were performed at D0, D56, and D84 by using the apparatus Life Viz mini, and volume measurements (mm^3^) were obtained by using image analyses from Life Viz micro photographs.

#### 2.3.5 IL8 and Hydrogen Peroxide Assays From D‐Squame Patches for Anti‐Inflammatory and Antioxidant Effects Respectively

D‐squame patches are a non‐invasive sampling method for the analysis of molecules present in the stratum corneum. The amount of hydrogen peroxide in the stratum corneum is an indicator of the state of the oxidation of the skin, while that of IL‐8 chemokine is an indicator of local inflammation. Samples were collected using 3.8 cm^2^ D‐squame patches (CuDerm) at D0, D56, and D84 on the left or right cheeks. Six patches were successively applied on the same area (the first patch was thrown out), and each of the 5 patches was placed in a tube containing 300 μL of tris–HCl buffer and 0.1% Triton X100 for IL‐8 quantification and of methanol for H_2_O_2_ measurements. After 20 min of cold sonication, the cells were eliminated by centrifugation at 7000 g for 10 min, and the 5 surpernatants obtained were pooled in another tube. The IL‐8 assay was performed after spectrophotometric reading (λ450 nm) (BioTechne kit) of these supernatants. The results were expressed in pg/ml. The hydrogen peroxide assay was performed by spectrocolorimetric analysis on the supernatants with a Hydrogen Peroxide (H_2_O_2_) Colorimetric assay Kit (EalbScience). Following Micro BCA Protein Assays (Thermo Fisher) on the supernatants, the final results were expressed in pg/μg protein for IL8 concentrations or in nmol/μg protein for H_2_O_2_ concentrations.

#### 2.3.6 Ultrasound Measurement of Dermal Density

The measurement was carried out from the images taken on the right cheek in contact with the nose by the DermaScan ultrasound apparatus (2D 20 Mhz probe; 12.1 mm of narrow‐focus exploration) using Advanced Control acquisition and analysis software. Areas in yellow showed high reflective strength (high density of tissues mainly made up of collagen), and areas in black show low density tissues. The increase in the percentage of high‐density areas (in yellow) indicated a stimulation of the production of collagen and, consequently, an increase in skin firmness. The interpretation scale was as follows: in black, 0‐ low ultrasound reflection representing low tissue density, and in yellow, maximum ultrasound reflection limit representing dense tissue.

The dermis density results were expressed in percentages calculated from the surface occupied by collagen in the dermis versus the total surface measured.

#### 2.3.7 Profilometry of a Target Crow's Feet Wrinkle

Profilometric evaluations of the volume (mm^3^) and the depth (mm) of a target crow's feet wrinkle were made using the apparatus LifeViz micro (three‐dimensional visualization). The depth and volume analyses were performed on the deepest wrinkle of the target zone (right or left).

#### 2.3.8 Evaluation of Skin Appearance With Dermatological Scorings

Skin radiance is a complex parameter that involves not only the quantity of light reflected from the skin but also physical aspects like skin color and skin relief. To evaluate skin radiance, the dermatologist used scores from 0 to 4 (score 0 corresponding to a luminous skin and 4 to a dull complexion) [27].

For the sagging of the lower part (oval) of the face, the BAZIN scoring (between 0 and 5, grade 5 corresponding to a very high skin laxity) was used to evaluate the extent of sagging of the lower part of the face.

#### 2.3.9 Safety and Tolerance

At D56 and D84 of the clinical trial, the dermatologist checked for the absence of adverse events. In case of an adverse event, a clinical investigation was conducted by the dermatologist, possibly with photographs, and, if necessary, a treatment prescription was given.

#### 2.3.10 General Assessment and Self‐Assessment

A self‐assessment questionnaire was given to each subject and was used to evaluate the efficacy of the product at D56 and D84.

#### 2.3.11 Statistical Analyses

All statistical analyses were performed with the GraphPad software. For in vitro studies, results were analyzed with an unpaired t‐test to compare each treatment condition versus its control condition. For the clinical study, the mean values and standard deviations of the different parameters at each time point of the study were determined for the 20 subjects. Comparisons of the data obtained at D56 and D84 to the initial values (D0) were performed. The Shapiro–Wilk test was used to check the normality of distributions between the right and left sides of the face. If normality of distribution was confirmed, the bilateral paired Student test (parametric test) was performed. Otherwise, the non‐parametric Wilcoxon test was performed. For all statistical tests performed, the significance threshold was *p* < 0.05.

## 3 Results

### 3.1 In Vitro Studies

#### 3.1.1 TFC‐1326 Preserves the Viability of Human Dermal Fibroblasts Cultured in Stress Conditions

**FIGURE 2:**
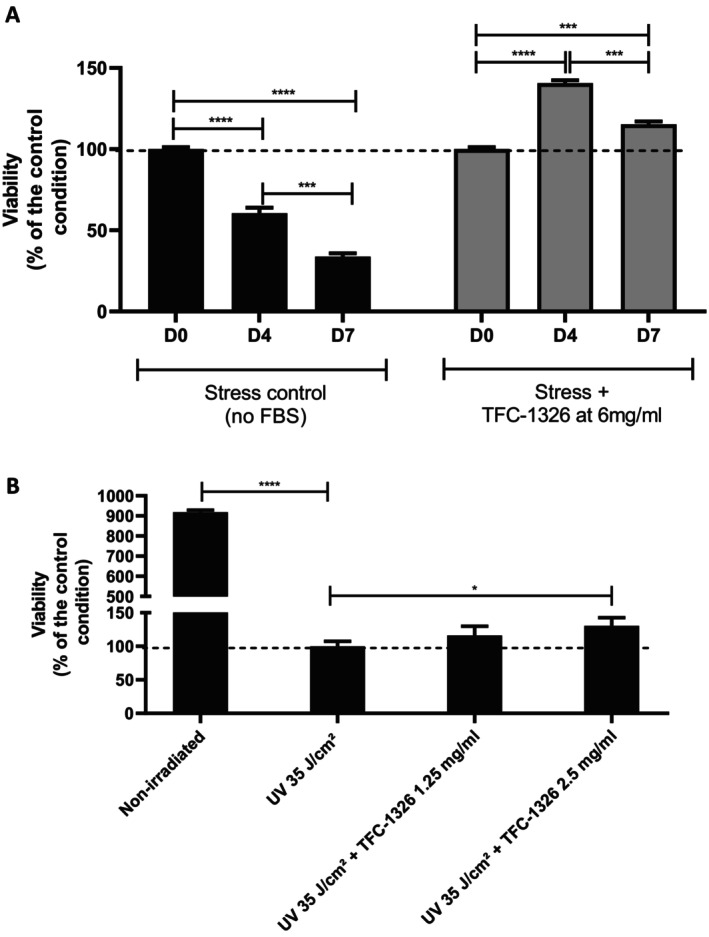
Evaluation of the effects of TFC‐1326 on two stress models of dermal fibroblasts. Cell viability was assessed in FBS‐depleted (A) or irradiated (B) conditions. Results are expressed as means ± SD. *p < 0.05, ***p < 0.001, ****p < 0.0001.

In a preliminary study, we assessed the efficacy of TFC‐1326 in two stress models. For that, we cultured dermal fibroblasts in medium without FBS for 7 days to induce a progressive decrease in cell viability. TFC‐1326, at a concentration of 6 mg/mL, was able to restore or even stimulate cell viability after 4 and 7 days of culture without FBS (Figure 2A). In the same way, the compound, at a concentration of 2.5 mg/mL, was notably able to increase the viability of fibroblasts, which had been strongly altered in irradiated conditions (Figure 2B). All together, these results revealed that TFC‐1326 can preserve cell survival in stress microenvironments.

#### 3.1.2 TFC‐1326 Reverses the Deleterious Effects of Inflammation on the Biology of Human Preadipocytes

After confirming a protective effect on cell survival, we set out to explore the potential of compounds in models mimicking different human skin conditions. According to preliminary cytotoxicity results (data not shown), we decided to test the concentrations of 1, 2, and 5 mg/mL of the compound in a model of human preadipocytes cultured in proadipogenic and proinflammatory conditions.

**FIGURE 3:**
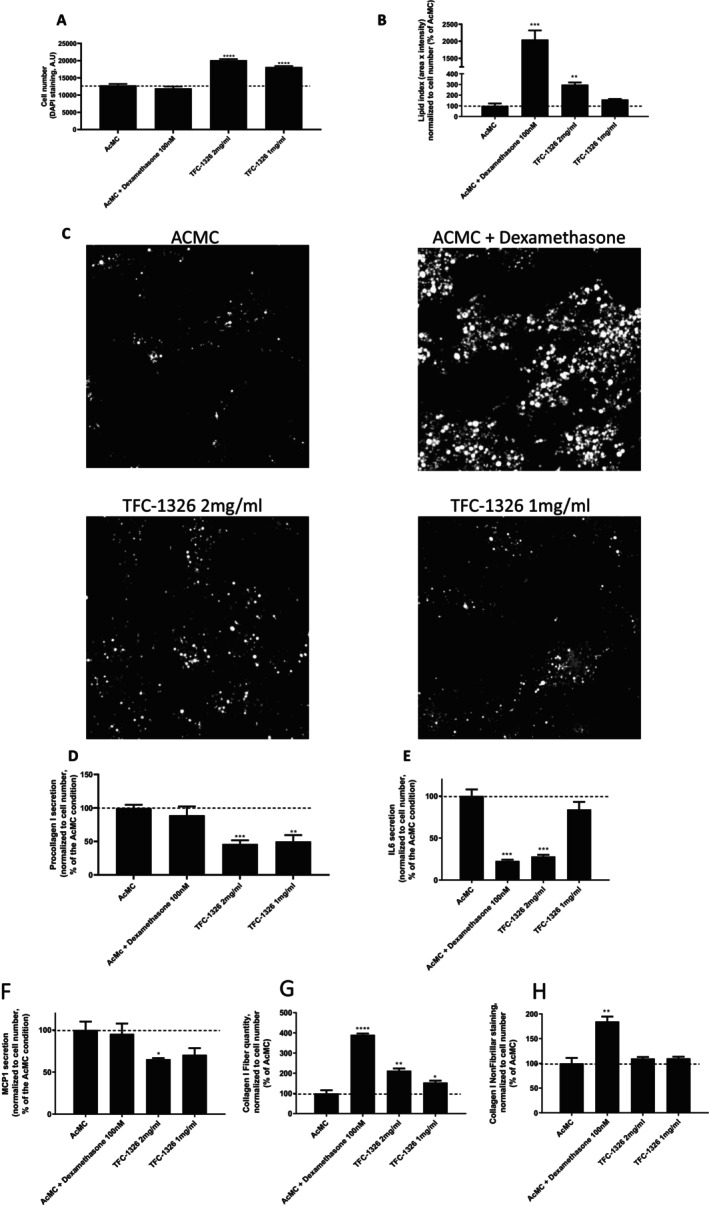
Evaluation of the effects of TFC‐1326 on human preadipocytes in proinflammatory conditions. Cell number (A) and lipid accumulation (B) were quantified after specific fluorescent staining. Representative microphotographs of lipid droplets in human preadipocytes (white staining) (C). Concentrations of procollagen I (D), IL6 (E) and MCP1 (F) were determined in the secretions of human preadipocytes. Fibrillar (G) and non‐fibrillar (H) type I collagen were quantified after immunostaining experiments. Results are expressed as means ± SD. *p < 0.05, **p < 0.01, ***p < 0.001, ****p < 0.0001.

The concentration of 5 mg/mL rapidly induced cell detachment, suggesting a cytotoxic effect of the product (data not shown) and was hence excluded from further studies. Whereas with concentrations of 1 and 2 mg/mL of TFC‐1326, we observed a higher number of preadipocytes than in the proinflammatory control, ACMC condition (Figure 3A), suggesting an increased proliferative capacity of the cells.

Moreover, as expected, the preadipocytes treated with the reference product, dexamethasone at 100 nM, largely accumulated more lipids than cells in ACMC alone. In addition, TFC‐1326, notably at 2 mg/mL, was also able to stimulate lipid accumulation in preadipocytes but to a lower extent than that of dexamethasone (Figure 3B and Figure 3C). The secretion by preadipocytes of the extracellular matrix protein, procollagen I, (Figure 3D), and of the proinflammatory molecule, MCP‐1, (Figure 3F), was substantially reduced by the two doses of the product without dose effect. The secretion of the proinflammatory cytokine, IL6 (Figure 3E), was strongly inhibited by the product at a concentration of only 2 mg/mL, that is to a similar level as the inhibitory effects seen by the anti‐inflammatory drug dexamethasone. The immunostaining experiments revealed that the product, like dexamethasone, increased the quantity of collagen I, in a significant and dose‐dependent way for the fibrillar collagen I (Figure 3G and Figure 3H).

#### 3.1.3 TFC‐1326 Modulates the Inflammatory State and Induces Extracellular Remodeling in a Coculture of Aged Dermal Fibroblast and Mature Adipocytes

**FIGURE 4:**
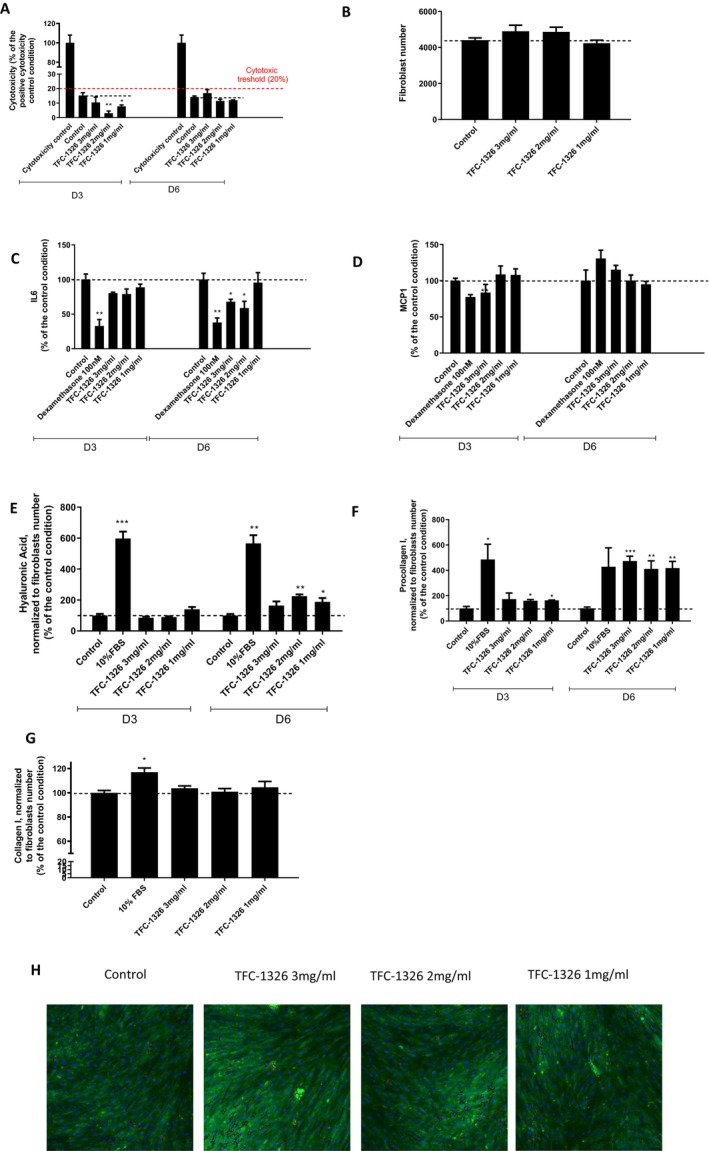
Evaluation of the effects of TFC‐1326 in a coculture of human mature adipocytes and dermal fibroblasts. Cytotoxicity (A) and fibroblast number (B) were assessed in the co‐culture. Concentrations of IL6 (C), MCP1 (D), procollagen I (E) and hyaluronic acid (F) were determined in the secretions of the co‐culture. Collagen 1 protein expression was quantified in fibroblasts after immunofluorescence staining (G). Representative microphotographs of collagen 1 network in fibroblasts after fluorescent staining (collagen 1 in green and cell nuclei in blue) (H). Results are expressed as means ± SD. *p < 0.05, **p < 0.01, ***p < 0.001, ****p < 0.0001.

After studying TFC‐1326 in a proinflammatory preadipocyte model, we decided to investigate its effects on a 6‐day coculture of aged human dermal fibroblasts and mature adipocytes, which are the main cells in the dermis and the hypodermis, respectively. As we observed a cytotoxic effect of TFC‐1326 at 5 mg/mL in preadipocyte experiments, consequently, we decided to use the dose of 3 mg/mL. As shown in Figure 4A, LDH activity remained below the cytotoxicity threshold for the three concentrations of TFC‐1326 used on D3 and D6 compared to control preadipocytes, suggesting no cytotoxic effects of the product at these doses. These results were confirmed by the fibroblast number, which increased at concentrations of 3 and 2 mg/mL of the compound compared to the control condition (Figure 4B), suggesting a positive effect of TFC‐1326 on the proliferative capacity of dermal fibroblasts.

Like dexamethasone, TFC‐1326 at the two highest concentrations was able to decrease the secretion of the proinflammatory cytokine, IL6, in the coculture at D3 and D6 (Figure 4C) and of the proinflammatory chemokine, MCP1, but only at D3 (Figure 4D). The product, at the 3 doses, tended to increase the secretion of hyaluronic acid at D6 (Figure 4E), whereas it strongly stimulated the production of procollagen I at D6, to the same level as the positive control, enriched with 10% of FBS (Figure 4F). Immunostaining experiments revealed a slight upward trend on the collagen I network of the dermal fibroblast (Figure 4G) after 6 days of culture with the product.

### 3.2 Clinical Assessment

All of the 20 subjects included in the study completed the protocol. All parameters were collected and analyzed for them.

#### 3.2.1 The Topical Cream Containing 1% TFC‐1326 Presents Anti‐Inflammatory and Antioxidant Effects on the Skin While Improving Its Radiance

**FIGURE 5:**
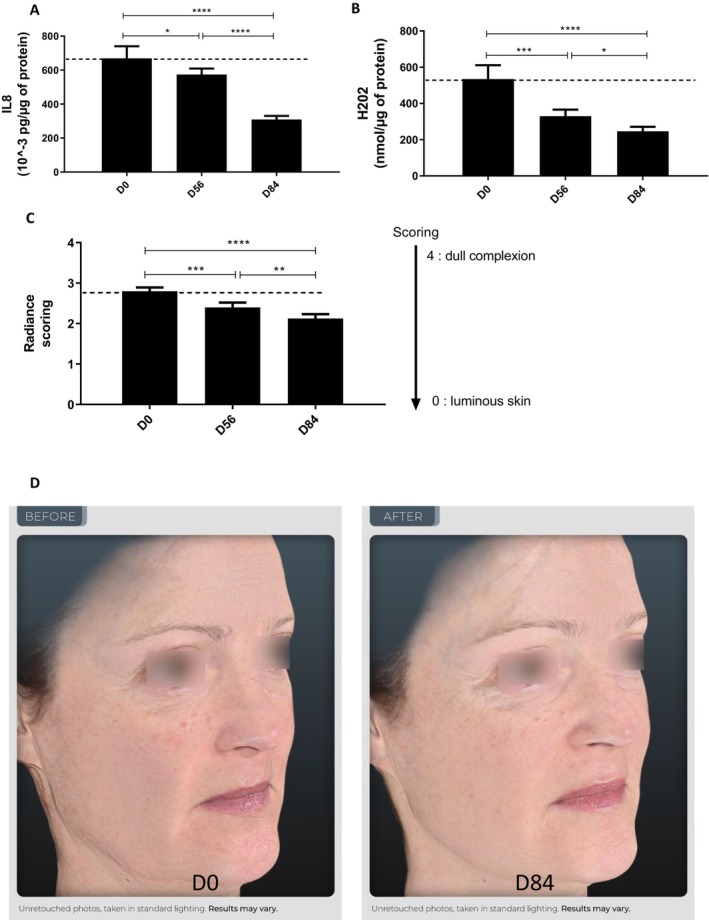
Clinical evaluation of a topical product with 1% of TFC‐1326 on inflammatory and oxidant states and on radiance of human skin. The concentrations of IL8 (A) and of hydrogen peroxide (H_2_O_2_) (B) were quantified in skin from D‐squame patches. Skin radiance was assessed by an internal dermatological scoring (C) (from 0 [luminous skin] to 4 [dull complexion]). Results are expressed as means ± SEM, *N* = 20. *p < 0.05, ****p < 0.0001. Representative photographs of skin radiance (D) from 1 woman (subject n°11) before (D0) and after (D84) the topical application with 1% TFC‐1326.

The concentrations of IL8 and of hydrogen peroxide (H_2_O_2_) from D‐squame patches applied on the face skin of subjects were significantly reduced with time after 56 and 84 days of the cream application. The level of IL‐8 was significantly decreased by 14.23% at D56 (0.668 ± 0.312 pg/μg at D0 vs. 0.574 ± 0.153 pg/μg at D56, *p* < 0.05) and by 53.8% after 84 days of cream application (0.309 ± 0.094 pg/μg at D84, *p* < 0.0001), revealing an anti‐inflammatory effect (Figure 5A). The level of H_2_0_2_ was significantly decreased by 38.3% at D56 (534.33 ± 333.02 nmol/μg at D0 vs. 329.66 ± 155.74 nmol/μg at D56, *p* < 0.001) and by 53.9% after 84 days of cream application (246.22 ± 107.29 nmol/μg at D84, *p* < 0.0001), supporting an antioxidant action (Figure 5B).

These anti‐inflammatory and antioxidant effects were associated with an improvement of the skin radiance determined by an internal dermatological scoring. Skin radiance was significantly improved by 14% at D56 (2.80 ± 0.40 A.U. at D0 vs. 2.40 ± 0.51 A.U. at D56, *p* < 0.001) and by 25% (2.13 ± 0.47 A.U. at D84, *p* < 0.0001) after 84 days of the 1% TFC‐1326 cream application (Figure 5C,D).

#### 3.2.2 The Topical Cream Containing 1% of TFC‐1326 Increases the Density of the Dermis and Improves Skin Laxity of the Face Oval

**FIGURE 6:**
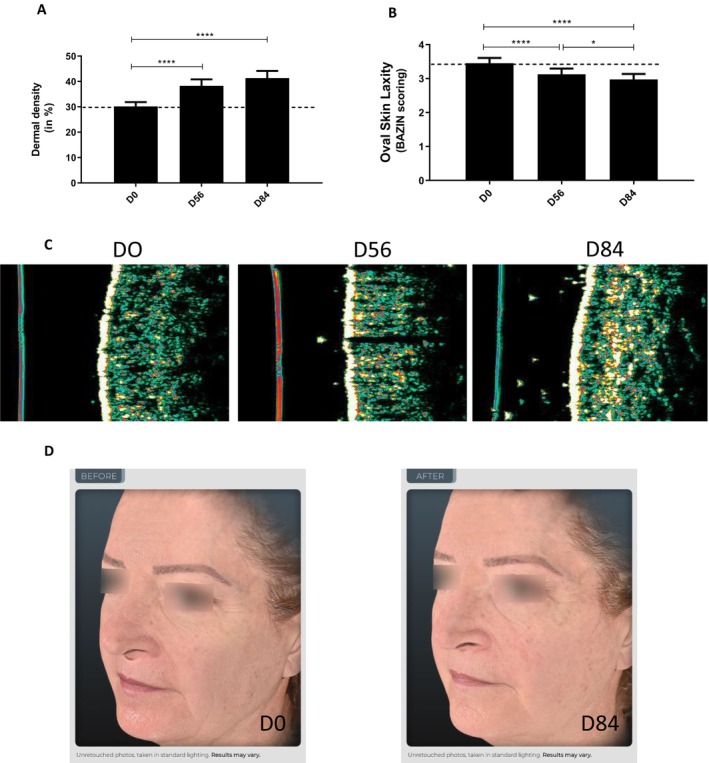
Clinical evaluation of the product with 1% TFC‐1326 on dermal density and skin laxity. Density of the dermis (A) was evaluated by ultrasound measurements, and skin laxity of the face oval (B) was assessed by Bazin scoring. Results are expressed as means ± SEM, *N* = 20. *p < 0.05, ****p < 0.0001. Representative ultrasound images of dermis density (C) and representative photographs of face sagging (D) from one woman (subject *n*°12) before (D0), during (D56), and after (D84) the topical application with 1% TFC‐1326.

As shown in Figure 6A,C, ultrasound measurements revealed a significant increase in the dermal density of the cheek by 26.84% at D56 (30.17 ± 7.33 A.U. at D0 vs. 38.26 ± 11.14 A.U. at D56; *p* < 0.0001) and by 36.94% after 84 days (41.31 ± 12.36 A.U. at D84; *p* < 0.0001) of the 1% TFC‐1326 cream application.

In the same way, the sagging of the facial oval as assessed by Bazin scoring was significantly decreased by 9.42% at D56 (3.45 ± 0.69 A.U. at D0 vs. 3.13 ± 0.74 at D56, *p* < 0.0001) and by 13.77% after 84 days of the application of the 1% TFC‐1326 cream (2.98 ± 0.70 A.U.; *p* < 0.0001) (Figure 6B). Representative photographs in Figure 6D illustrate the decreased laxity of the facial oval.

#### 3.2.3 The Topical Cream Containing 1% TFC‐1326 Improves the Appearance of Crow's Feet Wrinkles in Depth and in Volume

**FIGURE 7:**
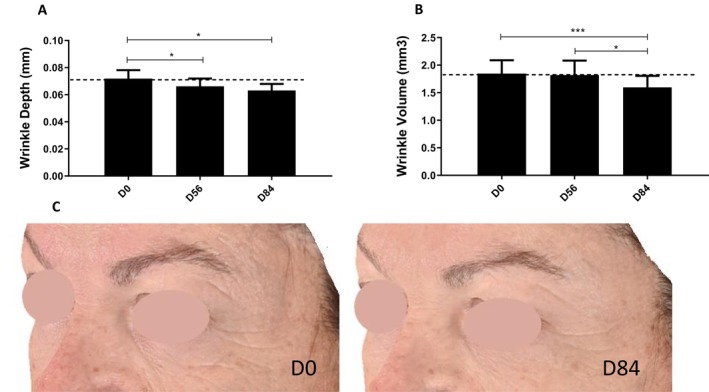
Clinical evaluation of the effects of the product with 1% TFC‐1326 on crow's feet wrinkles. Depth (A) and volume (B) of one wrinkle in the crow's feet area were determined by profilometric analysis. Results are expressed as means ± SEM, *N* = 20. *p < 0.05, ***p < 0.001. Representative photographs of the crow's feet area (C) from one woman (subject *n*°20) before (D0) and after (D84) the topical application with 1% TFC‐1326.

Profilometric evaluation revealed a significant decrease in crow's feet wrinkle depth by 7.86% at D56 (0.072 ± 0.027 mm at day 0 vs. 0.066 ± 0.025 mm at D56; *p* = 0.01) and by 12.18% at D84 (0.063 ± 0.021 mm; *p* = 0.01) (Figure 7A) and in its volume by 13.6% only on D84 (1.847 ± 1.055 mm3 at D0 vs. 1.597 ± 0.912 mm3 at D84; *p* < 0.0001) (Figure 7B).

#### 3.2.4 Global Assessment by Volunteers After 84 Days of the Compound Cream Application

An improvement of the skin radiance with a decrease of wrinkles was observed by 75% of the subjects. Of the 20 volunteer subjects, 80% felt that their skin was firmer with an increase in skin quality and hydration of 90%. The skin was felt to be smoother for 85% of the volunteers.

Dermatological tolerance was excellent for all the volunteers. No comedogenic effect was observed by volunteers or by the dermatologist throughout the 84 days of product application.

## 4 Discussion

In this article, we propose an approach to alleviating the undesirable effects of aging on skin. In particular, we investigated the potential properties and benefits of a new family of compounds based on anti‐freeze glycoproteins, known for their stress protective effects, and more specifically of one compound, TFC‐1326, for reducing the impact of aging on the cutaneous biology. Preliminary studies of models of stress induced by serum starvation or by irradiation highlighted that TFC‐1326 preserved the viability of human dermal fibroblasts and confirmed the interest to explore its effects on different study models of inflammation and cellular interactions within the skin.

In a first model of human preadipocytes cultured in the presence of proinflammatory factors secreted by human macrophages [24, 25], TFC‐1326 was able to reverse the deleterious effects of inflammation on the biology of these adipose cells by stimulating their lipid synthesis while increasing their proliferative capacities. Moreover, it decreased the fibroinflammatory state of these cells by reducing their secretion of procollagen I, interleukin 6, and the chemokine MCP1. On the other hand, we observed an increase in fibrillar collagen I in preadipocytes treated with TFC‐1326. The decrease in procollagen I concentrations observed in the last 24 h of secretion in the culture of the preadipocytes suggests that a major part of the secreted procollagen I can be converted into collagen. These effects of TFC‐1326 on matrix remodeling further support the action of the compound on the structure, organization, and architecture of the adipose tissue. This merits further investigation, more particularly by studying the expression of other matrix proteins like fibronectin, tenascin‐C, or lysil oxydase, which participate in collagen maturation by initiating the cross‐linking of fibers [28]. Moreover, we noted an increase in lipid accumulation as well as similar modulation of procollagen I secretion and collagen I network as seen in the treatment with dexamethasone, suggesting that the mechanism of action of TFC‐1326 could be similar to that of the synthetic glucocorticoid [29]. More generally, it was shown that the loss of peripheral fat associated with aging may result in part from a decline in cell proliferation and adipogenesis and that these defective processes could be linked to the local proinflammatory state of the subcutaneous adipose tissue [30]. Indeed, it is tempting to suggest that TFC‐1326, thanks to its anti‐inflammatory properties, could help prevent fat loss brought about by aging. However, the effect of TFC‐1326 on increasing the number of adipose progenitors can also be attributed to the direct action of the compound on the cell cycle. Indeed, preliminary transcriptomic studies performed on dermal fibroblasts unveiled the stimulation of the expression of genes involved in the cell cycle, like the Cyclin D1/Cd4 complex, which contribute to governing the cell cycle and its progression (data not shown).

In a novel coculture system of human mature adipocytes with aged human dermal fibroblasts, TFC‐1326 was shown to decrease IL6 and MCP‐1 secretion, confirming the anti‐inflammatory properties of TFC‐1326 previously described in the model of proinflammatory preadipocytes. Conversely, it stimulated fibroblast secretion of matrix proteins like hyaluronic acid and procollagen I, notably after 6 days of coculture with very marked effects for the last one. However, the analysis of the collagen I network in fibroblasts revealed an increasing trend of its protein expression, though not significantly so, probably due to the short duration of the coculture. We suggest that the procollagen I mostly produced by the fibroblasts at the end of the coculture did not have the time to organize into a fiber network. As in the preadipocytes model and supported by transcriptomic results, TFC‐1326 was shown to induce matrix remodeling. Consequently, it would be of great interest to carry out complementary investigations on the secretion/production of metalloproteases responsible for the degradation of the extracellular matrix (ECM) or of other matrix proteins, like fibronectin, known to accelerate spontaneous collagen assembly [31]. This improvement in ECM produced by dermal fibroblasts could result from the direct effects of the compound on the mechanisms of matrix synthesis, but it may also be due to indirect effects on the fibroblast microenvironment improved by reduced proinflammatory factors and modulated by the secretome of adipocytes. Indeed, some adipokines like adiponectin and leptin have been described to influence positively the functionality of fibroblasts, mainly their capacities for proliferation and matrix protein synthesis [32].

As for its stress‐protective effects, its proliferative and anti‐inflammatory properties, and its capacity to remodel the ECM, it would be tempting to explore the potential efficiency of TFC‐1326 in cell models of wound healing [33] but also of senescence, which is known to be associated with aging and to be characterized by morphological and metabolic changes, chromatin reorganization, altered gene expression, and adoption of a specific proinflammatory phenotype described as the senescence‐associated secretory phenotype (SASP) [34]. Senescent cells and inflammation are indeed closely associated and contribute to each other's increase [35]. The ability of TFC‐1326 to reduce inflammation could contribute to reducing the generation and spread of senescent cells.

We also tested TFC‐1326 in a topical formulation and evaluated it through a pilot clinical trial on 20 women that showed encouraging results. The outcome of 3 months of continual application of the cream was increased skin radiance, reduced facial oval sagging, a decrease in the depth and volume of the crow's feet, drops in IL8 and H_2_O_2_ as well as improved dermal density. These clinical observations confirmed that the anti‐inflammatory power, together with the firming, remodeling, and anti‐wrinkles benefits of the TFC‐1326 cream, can be mechanistically explained through the preclinical effects seen on human adipose and skin cell models. Potentially related to its inhibitory actions on inflammation, this clinical study unveiled an antioxidant activity of TFC‐1326 that supported the detoxifying benefits of the compound. Biologically, this effect can also be linked to its stimulatory action on lipid synthesis in irradiated keratinocytes as well as its effects on preventing lipid peroxidation (data not shown).

These clinical data highlight the need for further studies to confirm these findings in a larger population and to compare with a placebo formula. Moreover, given its anti‐inflammatory properties, TFC‐1326 may represent a potential candidate for the treatment of more pathological skin conditions like atopic dermatitis that merit to be further explored.

In conclusion, supported by a large preclinical investigation performed in predictive models of skin biology and a first clinical study, the synthetic compound, TFC‐1326, derived from the anti‐freeze glycoproteins family, proves to be an original and promising cosmetic active agent for reducing the visible signs of aging in the skin.

## Author Contributions

Conceptualization, G.D.‐G. S.B., and M.K.; methodology, S.B., L.‐T.N., and M.K.; software, S.B. and M.K.; validation, S.B. and M.K.; formal analysis, M.‐C.B., S.B., M.B., and M.K.; investigation, S.B.; resources, S.B. and M.K.; Visualization: G.D.‐G. J.L., M.B., N.J., and M.K.; writing – original draft preparation, G.D.‐G., J.L., M.B., and M.K.; writing – review and editing, G.D.‐G., J.L., and M.K.; supervision, S.B. and M.K.; project administration, S.B. and M.K.; All the authors read and approved the article.

## Ethics Statement

The monocentric study was conducted in accordance with Good Clinical Practices and the principles of the Declaration of Helsinki. According to local regulatory guidelines, this type of trial testing in cosmetics did not require approval from local ethics committees. Subjects provided written informed consent prior to participation.

## Conflicts of Interest

M.K., S.B., M.B., N.J., L.T.N., and M.C.B. have no conflict of interest to disclose. GDG and JL are employees of TFChem (Sirona Biochem Group). The funding sponsors had no role in conducting or monitoring the preclinical and clinical studies; nor in the collection, analysis or interpretation of the data.

## Data Availability

The data that support the findings of this study are available on request from the corresponding author. The data are not publicly available due to privacy or ethical restrictions.
